# Therapeutic Prospects of αv Integrins Inhibition in Fibrotic Lung Diseases and Carcinogenesis

**DOI:** 10.3390/ijms26136202

**Published:** 2025-06-27

**Authors:** Eugenija Leonidovna Golovina, Veronika Vladimirovna Kochubey, Marina Alekseevna Shabanova, Darya Maksimovna Chekhvalova, Valentina Alexandrovna Serebryakova, Evgenii Germanovich Skurikhin, Olga Evgenievna Vaizova, Sergey Georgievich Morozov, Aslan Amirkhanovich Kubatiev, Alexander Mikhaylovich Dygai

**Affiliations:** 1 Department of Pharmacology, Siberian State Medical University, Ministry of Health of the Russian Federation, Tomsk 634050, Russia; golovina.el@ssmu.ru (E.L.G.); shabanova.ma54@gmail.com (M.A.S.); chekhvalovadarya@mail.ru (D.M.C.); serebryakova.va@ssmu.ru (V.A.S.); vaizova.oe@ssmu.ru (O.E.V.); 2 Laboratory of Regulation of Reparative Processes, Institute of General Pathology and Pathophysiology, Moscow 125315, Russia; eskurihin@inbox.ru (E.G.S.); smorozov.biopharm@mail.ru (S.G.M.); akubatiev.niiop@mail.ru (A.A.K.); amdygay@gmail.com (A.M.D.); 3 Laboratory of Regulation of Reparative Processes, Goldberg Research Institute of Pharmacology and Regenerative Medicine, Tomsk National Research Medical Center, Russian Academy of Sciences, Tomsk 634050, Russia

**Keywords:** αv integrin, idiopathic pulmonary fibrosis, chronic obstructive pulmonary disease, lung cancer

## Abstract

The uncontrolled fibrosis of lung tissue can lead to premature death in patients suffering from idiopathic pulmonary fibrosis (IPF), and it complicates the course of chronic obstructive pulmonary disease (COPD) and emphysema. It is also a risk factor for developing lung cancer. Antifibrotic drugs, such as nantedanib and pirfenidone, are able to slow down the progression of pulmonary fibrosis, but more effective treatment is still needed to reverse it. Studies on the pathogenesis of tissue fibrosis have demonstrated that integrins play a crucial role affecting the development of pulmonary fibrosis, for example, by activating transforming growth factor-β (TGF-β). Taking the above into consideration, targeting specific integrins could offer promising opportunities for managing fibroplastic changes in lung tissue. Integrins are a type of transmembrane molecule that mediate interactions between cells and extracellular matrix (ECM) molecules. This review discusses the role of integrins in the pathogeneses of respiratory diseases and carcinogenesis, as well as presents promising approaches to the drug therapy of pulmonary fibrosis of various etiologies based on integrin inhibition.

## 1. Introduction

Fibrotic lung diseases are a diverse group of disorders that involve the excessive deposition of extracellular matrix (ECM) components in the lungs, leading to scarring and the impairment of the lung function. This process can result in the progressive loss of lung elasticity and, ultimately, respiratory failure. The pathogeneses of fibrotic lung diseases include epithelial damage, inflammation, and the dysregulation of repair mechanisms. Idiopathic pulmonary fibrosis (IPF) directly relates to these groups [1].

Chronic obstructive pulmonary disease (COPD) is not typically classified as a fibrotic lung disease. However, there are similarities in the pathological processes involved in the pathogeneses of both conditions, particularly during the stage when respiratory tract remodeling and lung parenchymal changes occur, leading to the development of emphysema. At this stage, the repair processes and formation of fibrous tissue become more prominent, raising the possibility of considering the late stages of COPD to be fibrotic [2].

According to the World Health Organization (WHO), non-infectious respiratory diseases, particularly COPD, are among the leading causes of mortality in the population. IPF is another irreversible progressive disease of unknown etiology that is associated with a high mortality rate. Inflammation and oxidative stress play a key role in the pathogeneses of COPD and IPF, causing damage to the alveolar epithelium, the infiltration of the airway epithelium by inflammatory cells, fibroblast proliferation, and the excessive deposition of ECM components. Both diseases are risk factors for lung cancer due to multiple mechanisms of fibrosis that simultaneously act as elements of tumor growth pathogenesis. In COPD and IPF, the mechanisms of fibrotic cell transformation arise from the activation of transforming growth factor-β (TGF-β), which has also been shown to play a role in the development of lung cancer [3]. Managing uncontrolled fibrosis may be a strategy to prevent lung cancer, but currently there are only two available antifibrotic drugs, nintedanib and pirfenidone, which slow the disease progression [4]. Nintedanib is an intracellular tyrosine kinase inhibitor that exhibits antifibrotic and anti-inflammatory effects. Pirfenidone, a small molecule with antifibrotic, anti-inflammatory, and antioxidant effects, suppresses TGF-β production, myofibroblast proliferation and differentiation, and collagen deposition [5]. The drugs prevent reductions in the forced vital capacity and prolong the survival of patients with IPF, although they do not reverse cell transformation [4]. In phase II and III clinical trials, the efficacy and safety of monoclonal antibodies against the connective tissue growth factor, autotaxin-1 inhibitor, and zinpentraxin-alpha (human recombinant pentraxin-2), which seemed promising in the experiment, were not confirmed [6]. No effect of these drugs on lung cancer development has been reported. Therefore, the challenge of searching for and developing a new agent that targets fibrosis while preventing tumor growth is urgent.

Heterodimeric transmembrane receptors of the integrin family play a significant role in the process of fibrotic cell transformation. Currently, there are several ongoing developments of integrin inhibitor compounds, some of which have reached phase IIb or phase III clinical trials.

This narrative review aims to analyze information on the involvement of integrins in the pathogeneses of diseases associated with fibrotic remodeling in lung tissue and the potential use of integrin inhibitors to prevent these changes and reduce the risk of carcinogenesis.

## 2. General Information About Integrins

Integrins are heterodimeric transmembrane receptors that interact with the extracellular matrix (ECM) and transmit intercellular signals. Integrin-mediated interactions between cells and the ECM are involved in regulating various cellular processes, such as inflammatory responses, angiogenesis, migration, cell proliferation and differentiation, and gene expression. To date, 24 distinct integrins have been identified in mammals, which are combinations of 18 α and 8 β subunits [7]. Integrin molecules bind to specific ECM ligands through their large ectodomain. Binding to cytosolic ligands is mediated through an internal fragment (tail) via a single-spanning helical transmembrane domain [8]. Most integrin heterodimers contain αv and β1 subunits [9]. Integrins are divided into four classes based on their ligand-binding properties: integrins that bind the tripeptide motif arginyl–glycyl–aspartic acid (RGD), collagen-binding integrins, laminin-binding integrins, and leukocyte-specific integrins that bind to intercellular adhesion molecules, polysaccharides, and plasma proteins [10]. Heterodimeric complexes of integrins alter their affinities for ligands depending on the spatial arrangement of the extracellular domain sites relative to one another. The curved–closed conformation is unable to interact with the ligand, while the open conformation has maximum affinity for the ligand [9].

**Figure 1:**
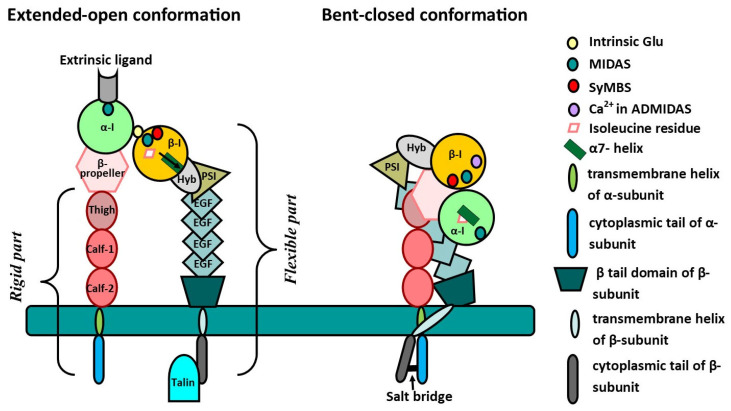
Structures of integrins’ α and β subunits. α-I—inserted domain of α subunit; β-I—inserted domain of β subunit; Hyb—hybrid domain; PSI—plexin–semaphorin–integrin; EGF—epidermal growth factor-like domain; Thigh—thigh domain of α subunit; Calf-1 and-2—Calf-1 and -2 domains of α subunit; Intrinsic Glu—intrinsic glutamic acid molecule; MIDAS—metal ion-dependent adhesion site; ADMIDAS—adjacent to MIDAS site; SyMBS—synergistic metal ion-binding site.

The α and β subunits are composed of several parts, including the extracellular segment, a single transmembrane helix, and usually a short, unstructured cytoplasmic tail (Figure 1) [11].

The α subunit is presented by four extracellular domains: a seven-bladed β-propeller with an inserted I domain, a “thigh”, and two “calf” domains. The active center of integrins is located in the I domain, which tends to be identical for both α and β subunits [12]. Only 7 of the 18 α subunits of integrins possess an α-I domain inserted between blades 2 and 3 of the β-propeller, while all β subunits have it [9]. It is significant to notice that the C-terminal α-helix of the I domain contains an invariant isoleucine residue, which is deeply embedded in the hydrophobic part of the α7-helix, thereby preventing the helix from moving down easily. The invariant isoleucine serves as an important intrinsic structural component to maintain a default low-affinity I domain [13]. The cytoplasmic tail of α subunits contains the GFFKR motif (Glu-Phe-Phe-Lys-Arg), the arginine of which makes a salt bridge with acidic residue (aspartate or glutamate) at the proximal region of the β subunit’s cytoplasmic domain [9].

The structure of α-subunit domains is relatively rigid, except the I domain, which is inserted into the β-propeller via flexible links and contains a metal ion-dependent adhesion site (MIDAS) motif, the major ligand-binding site [14].

The ectodomain of the β subunit, in turn, has a more complex and flexible structure compared to that of the α subunit. This includes a similar I domain that is inserted in the hybrid domain. Next, there is the plexin–semaphorin–integrin (PCI) domain, followed by four epidermal growth factor (EGF)-like domains and a β-tail domain [9]. The inserted domain contains three divalent metal ion-binding sites. The central site, similar to the α-I domain, known as the MIDAS, has Mg^2+^ and directly coordinates the side chain of an acidic residue found in integrin ligands, while the two outer sites adjacent to the MIDAS can also bind Mn^2+^, Mg^2+^, and Ca^2+^ [15]. One of these adjacent sites (ADMIDAS) coordinates an inhibitory Ca^2+^, thereby contributing to the stabilization of the C-terminal helix of the β subunit in the low-affinity configuration [13]. The second Ca^2+^-binding site has been called the synergistic metal ion-binding site (SyMBS), and it has been demonstrated that the presence of Ca^2+^ here promotes the enhancement of the MIDAS metal ion’s electropositivity and its ability to bind a ligand’s carboxyl oxygen [16]. Studies have demonstrated that when metal ions are removed from the MIDAS through residue mutations, the ligand binding to integrins is impaired. This suggests that the MIDAS plays a critical role in the coordination and binding of ligands [17]. All β subunits have distinct structural features, including the locations of the MIDAS and ADMIDAS, and are presented in Table 1.

The activation of integrins occurs inside the cell, when activated talin binds to the cytoplasmic NPxY motif (Asn-Pro-x-Tyr) of the β subunit (Figure 1). This interaction leads to the disruption of the salt bridge between the cytoplasmic tails of the two subunits, causing the β subunit to move relative to the alpha subunit. Consequently, due to the movement of the α7-helix along the hybrid region of the β subunit, the active site of the I domain becomes exposed, allowing an internal ligand (glutamate) to bind. This binding event triggers the opening of an active site on the α subunit that can then interact with an external ligand [13]. However, in cases when the I domain is not presented on the α subunit, after binding with talin, the activated β subunit can directly interact with the external ligand without requiring the involvement of the α subunit’s active site [18].

## 3. Interaction Between Integrins and TGF-β in Fibrotic Diseases and Cancer

Arginyl–glycyl–aspartic acid (RGD) binding αv integrins are abundantly expressed on the cell membrane in fibrotic foci and on cells of various tumors, including non-small-cell lung cancer (NSCLC), neuroblastoma, gastric carcinoma, and head and neck squamous cell carcinoma. They also appear to be involved in the pathogeneses of neurological and cardiovascular disorders [19].

**Figure 2:**
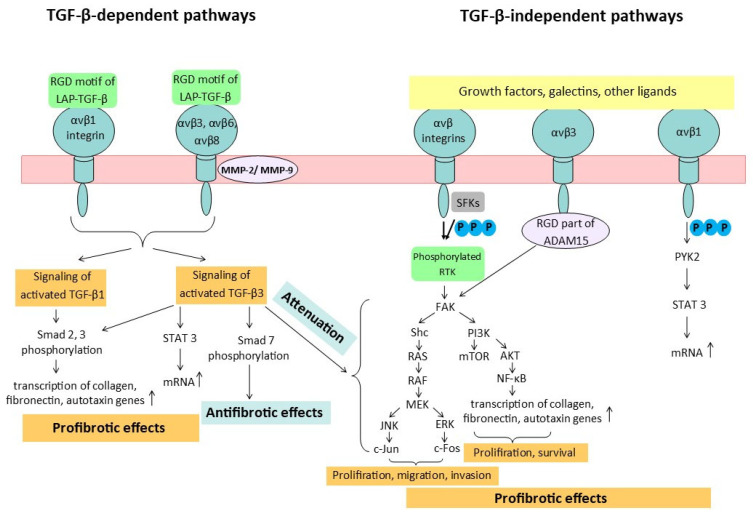
Integrin-associated fibrotic and oncogenic pathways. TGF-β—transforming growth factor-β; MMP-2—matrix metalloproteinase 2; MMP-9—matrix metalloproteinase 9; P—phosphoric acid residue; SFKs—SRC family kinases; RTK—receptor tyrosine kinase; FAK—focal adhesion kinase; ERK—extracellular signal-regulated kinase; PI3K—phosphatidylinositol 3-kinase; AKT—protein kinase B; mTOR—mammalian target of rapamycin; PYK2—proline-rich tyrosine kinase 2; STAT—activator of transcription; RGD—arginyl–glycyl–aspartic acid; ADAM15—metalloproteinase 15.

Transforming growth factor-β1 (TGF-β1) is a major contributor to the pathogenesis of fibrosis. The activation of TGF-β from its latent form requires a number of factors, in particular, the involvement of integrins. TGF-β is secreted in the form of inactive complexes with latency-associated peptides (LAPs). The activation of latent TFG-β1 and TFG-β3 isoforms occurs through two major pathways (Figure 2). The first pathway involves the recognition by αv integrins of the RGD motif as part of the LAPs. This recognition triggers the αvβ6 integrin to change the conformation of TFG-β by the mechanical force of the cell actin cytoskeleton, releasing TFG-β from its latent complex with LAPs. The second pathway of TGF-β activation occurs through the proteolytic degradation of its latent complex by matrix metalloproteinases (MMP-2 and MMP-9), although this process also requires the involvement of αv integrins. The induction of LAP conformational changes and sequestering the proteases in close proximity to LAPs in the latent complex of TGF-β1 occurs when αvβ6 and αvβ3 integrins bind to TGF-β1 and MMP simultaneously. The activation of TGF-β1 by αvβ8 integrins takes place only in a protease-dependent manner because the β8 subunit has a shortened cytoplasmic tail that is unable to bind to cytoskeletal components. Conversely, αvβ1 integrins directly bind to the LAP fragment of TGF-β1 and facilitate its activation [10]. TGF-β1, in turn, regulates multiple processes, including cell proliferation, angiogenesis, epithelial–mesenchymal transition, and immune suppression, all of which promote tissue regeneration. However, if left unregulated, these processes can lead to fibrosis and carcinogenesis [20].

While the involvement of the activated TGF-β3 isoform in the pathogenesis of fibrosis remains not entirely clear, studies have shown that fibrosis induced by TGF-β3 activation has similar properties but is less severe than the pronounced fibrotic effects of TGF-β1. Nevertheless, there is experimental evidence that TGF-β3 may accelerate wound healing with a reduced fibrotic response. Meanwhile, the use of recombinant human TGF-β3 as a potential inhibitor of hypertrophic scar formation in wounds has proven ineffective. It has been suggested that the specific effects of TGF-β1 and TGF-β3 isoforms may be related to their distinct patterns of localization and interaction with extracellular matrix (ECM) components, which, in turn, influence their binding to TGF-β receptors [21]. Experiments with the overexpression of TGF-β3 in rat lungs confirmed its profibrotic role, but the changes in the lungs were much weaker than those caused by TGF-β1. TGF-β3 does not inhibit matrix degradation in the same way as TGF-β1, which promotes non-fibrotic tissue repair. The profibrotic effect of TGF-β1 and TGF-β3 was expected to be associated with the increased phosphorylation of Smad 2 and 3 proteins. However, studies on other intracellular pathways have shown some differences in the action of TGF-β isoforms. TGF-β1 causes the stronger expression of the TGF-β type I receptor ALK 5 (Activin-like kinase 5) and the TGF-β type II receptor compared to TGF-β3 at both the gene and protein levels. Ask et al. suggest that an increased ratio of TGF-β I receptors to TGF-β II receptors may contribute to fibrotic degeneration, and that TGF-β isoforms have different effects on this ratio [22].

The primary reason why TGF-β3 exhibits a dual effect in various tissues is due to the presence of different signaling pathways, namely, the canonical and non-canonical signaling pathways. The canonical pathway, also known as the Smad-dependent pathway, is associated with fibrosis. When the Smad 2 and 3 signaling pathway is activated, it triggers the transcription of genes involved in ECM formation, such as collagen and fibronectin. This process contributes to the development of fibrosis in tissues [23].

The profibrotic role of TGF-β3 has been confirmed in cultured cells of the human eye trabecular reticulum. The autotaxin expression was significantly increased in trabecular meshwork cells treated with low concentrations of TGF-β3, corresponding to the physiological level. It was demonstrated that autotaxin transactivation occurred at least partially through the canonical Smad 3 pathway, as well as the non-canonical STAT3 and SAPK/JNK pathways, leading to fibrotic changes [23].

At the same time, there is some experimental evidence to suggest that TGF-β3 may play an important role in preventing fibrosis in corneal cells, skin wounds, and cardiac fibroblasts. The role of TGF-β1 and TGF-β3 in corneal fibrosis development was investigated using a 3D model composed of rabbit corneal fibroblasts treated with 0.1 ng/mL TGF-β1 or TGF-β3, as well as an ex vivo model of wounded rabbit corneas allowed to heal in culture with 1.0 ng/mL TGF-β1 or TGF-β3. It was found that TGF-β3, at different concentrations, compared to TGF-β1, decreased the expression of α-smooth-muscle actin mRNA in both the ex vivo organ culture and in vitro 3D cellular models, leading to a reduction in corneal scar formation [24].

The study of the effects of TGF-β1 and TGF-β3 on human corneal fibroblasts revealed an increase in the expression of certain genes, including type I collagen (*COL1*), Smad 2 and 3, and thrombospondin-1 (*THBS1*), during the early stages of healing. With prolonged exposure, differences in the effects of these two factors on the gene expression associated with fibrosis became apparent. It was found that TGF-β3 significantly increased the expression of Smad 7, an intracellular antagonist of the TGF-β/Smad signaling pathway, and it therefore stimulated the accumulation of a non-fibrous matrix in corneal fibroblasts [25].

Non-canonical signaling pathways (MAPK, PI3K/AKT, JNK, etc.) may modulate or attenuate profibrotic effects. TGF-β3 appears to be more involved in regulating the balance between these signaling pathways rather than actively stimulating fibrosis, as in the case with TGF-β1. For example, renal TGF-β3 may exhibit antifibrotic and renoprotective properties by counteracting or neutralizing the effects of TGF-β1 [26].

The increased phosphorylation of Smad 2 and 3 proteins, which are key components of the canonical TGF-β signaling pathway, was observed in normal dermal fibroblasts and resting scar fibroblasts in the study of Urban L. et al. Conversely, decreased signaling was demonstrated in keloid and active/hypertrophic scar fibroblasts after treatment with TGF-β3. This suggests that TGF-β3 may contribute to wound healing without the formation of scar tissue [27].

TGF-β3 decreased the proliferation, migration, and collagen synthesis in a culture of human cardiac fibroblasts by modulating the TGF-β/Smad signaling pathway, which may be related to the regulation of Smad 7. Thus, TGF-β3 prevented myocardial remodeling after a heart attack (Figure 2) [28].

The functional role of TGF-β2 is primarily the regulation of cellular growth, proliferation, migration, and differentiation during embryonic development (especially in the cardiovascular and immune systems). TGF-β2 activation does not require integrins, since the LAP fragment of TGF-β2 lacks the RGD motif [29].

Therefore, the use of systemic inhibitors of TGF-β1 or TGF-β3 as targets for fibrotic therapy is not feasible due to potential side effects. TGF-β1 and TGF-β3 are involved in normal cellular proliferation and tissue regeneration during homeostasis. At the same time, αv integrins are overexpressed in fibrotic tissues, leading to the local activation of TGF-β, which will be discussed in the next section. As a result, targeting αv integrins is an attractive therapeutic approach for fibrosis-associated diseases.

## 4. Integrin-Mediated Fibrosis and Carcinogenesis Mechanisms

Integrins are transmembrane complexes that maintain the tissue integrity, intercellular interactions, and cell response to extracellular matrix (ECM) changes, as well as the reverse reaction of ECM changes to internal changes occurring in cells. According to this role, integrins are involved in various pathological processes associated with alterations in cellular activity and changes in the ECM structure [30].

Of the 24 known heterodimers of α/β integrins, 12 contain the β1 subunit. β1 integrins are expressed on the membranes of epithelial cells, mediating their adhesion to the basal membrane and epithelial tissue growth [7]. The presence of β1 integrins in type 2 alveolar epithelial cells is associated with a pathology similar to that of chronic obstructive pulmonary disease (COPD). Inflammation and emphysema-like remodeling are observed in the lungs of adult mice with the altered function of type 2 alveolar epithelial cells lacking β1 integrins. This is associated with the increased production of reactive oxygen species and elevated levels of certain chemokines, particularly C-C motif chemokine ligand 2 (CCL2), and occurs by the nuclear factor kappa-light-chain-enhancer of activated B cells (NF-kB) [31]. The inhibition of the αvβ1 integrin has been shown to attenuate bleomycin-induced pulmonary fibrosis. The authors demonstrated that the synthesized integrin αvβ1 inhibitor, designated as c8, administered subcutaneously to C57BL/6 mice with bleomycin-induced pulmonary fibrosis, reduced the total amount of collagen and hydroxyproline in the lung tissue, as well as inhibited Smad fluorescence. However, the dose of the experimental inhibitor and its administration schedule were not specified [30].

The only subtype of integrin that is formed by the β6 subunit is the αvβ6 integrin [7]. Normal adult tissues lack the αvβ6 integrin, but it is significantly increased in damaged tissues and malignant tumors. In healthy lung tissue, epithelial cells express αvβ6 at a negligible level [32]. However, in models with idiopathic pulmonary fibrosis (IPF), alveolar epithelial cells overexpress αvβ6 integrins compared to healthy representatives [33]. The αvβ6 integrin contributes to the development of pulmonary fibrosis by activating transforming growth factor-β (TGF-β) [34]. It converts TGF-β1 and TGF-β3 into their active forms in a protease-independent manner through binding to the arginyl–glycyl–aspartic acid (RGD) motif in the LAP structure of latent TGF-β [35]. Activated TGF-β induces the profibrogenic differentiation of myofibroblasts [36] and promotes the fibrosis of the lung, liver, kidney, and heart, as well as systemic sclerosis [37]. In contrast, in a mouse model of intestinal fibrosis, it was observed that the αvβ6 integrin mediated epithelial cell degeneration through a focal adhesion kinase (FAK)/protein kinase B (AKT) signaling cascade rather than via the TGF-β signaling pathway. Western blot analysis has demonstrated a high concentration of the phosphorylated form of FAK in the intestinal tissues of transgenic mice with overexpressed β6 integrins [38]. Increased levels of αvβ6 integrins have been described in various fibrotic diseases, but their involvement in the pathogenesis of COPD is uncertain. In transgenic mice, the absence of αvβ6 integrins induced by the deletion of the *ITGB6* gene encoding the β6 subunit results over time in macrophage-rich inflammation and spontaneous emphysema, effectively reproducing an experimental model of COPD. In this model, deficiency in αvβ6 integrins leads to the formation of age-related emphysematous changes by the enhanced expression of macrophage metalloelastase (MMP12). The development of emphysema was prevented when MMP12 was blocked and the full-length human β6 integrin subunit was administered to transgenic mice. The reduced expression or function of αvβ6 disrupts the TGF-β signaling pathway [39]. The role of TGF-β in the context of COPD needs to be clarified. Its complete absence can slow down inflammation progression, but excessive levels of TGF-β can lead to respiratory fibrosis [40]. These findings suggest that although αvβ6 integrins and TGF-β are implicated in COPD-related mechanisms, their interaction is complex, and the absence of αvβ6, rather than its overexpression, leads to emphysema in experimental models.

It should be highlighted that the overexpression of integrin αvβ6 associated with IPF has been described in humans, while emphysematous changes associated with deficiency have been described in mice, so this paradox can be partially explained by species differences in the expression or functional role of integrin αvβ6. However, the phenomenon that has been identified certainly needs to be studied in more depth, and the maximal concentration of efforts is probably required to explain this paradox, as well as developing a strategy to avoid this side effect if it does occur in representatives of the same biological species.

The activation of TGF-β by αvβ8 integrins in COPD promotes the profibrogenic differentiation of fibroblasts. The increased expression of αvβ8 integrins in the airway fibroblasts of COPD patients directly correlates with the degree of airway wall fibrosis [41].

Formerly known as the vitronectin receptor, the αvβ3 integrin interacts with a variety of other ligands: fibronectin, Willebrandt’s factor, laminin, and osteopontin. Its enhanced expression has been observed on vascular endothelial cells in tumors of various types. Apart from angiogenesis, tumor cell invasion and metastasis are also dependent on αvβ3 integrins [10]. Thus, the involvement of TGF-β activated by its interaction with αv integrins in the development of pulmonary fibrotic diseases is confirmed.

In an experimental study, Giménez et al. noticed that the stiffness of the ECM increases the sensitivity of integrins to mechanical signals in lung fibroblasts by phosphorylating FAK at Tyr-397. This, in turn, activates AKT, which leads to the activation of NF-κB. Activated NF-κB affects the transcription of genes involved in ECM synthesis (Figure 2) [42]. Activating FAK has several effects. Firstly, it promotes the migration of lung fibroblasts by binding to integrin β1 and ECM proteins. Secondly, it activates the Rho family of proteins, which additionally promotes fibroblast migration. Thirdly, it phosphorylates extracellular signal-regulated kinase (ERK), which enhances the proliferation of pulmonary fibroblasts and their differentiation into myofibroblasts. This leads to increased ECM secretion, contributing to the progression of IPF [43].

The role of integrins in the pathogeneses of respiratory diseases is not limited to IPF, COPD, and emphysema. Additionally, integrins potentiate carcinogenesis in lung tissue, as they regulate the phosphorylation of proline-rich tyrosine kinase 2 (PYK2) in non-small-cell lung cancer (NSCLC). Specifically, the αvβ1 integrin promotes the phosphorylation of PYK2 at the Tyr402 site in NSCLC, which, in turn, phosphorylates the activator of transcription 3 (STAT3). This leads to the transport of its active form, p-STAT3 (Tyr705), into the nucleus and increases the mRNA expression of the VGF nerve growth factor inducible, essential for regulating energy homeostasis and cell metabolism, which contributes to NSCLC progression [44]. The αvβ6 integrin enhances the ability of tumor cells to infiltrate the fibronectin-rich matrix surrounding NSCLC cells and promotes tumor progression and invasion through the TGF-β signaling pathway [45]. The concurrent high expression of disintegrin metalloproteinase 15 (ADAM15) further enhances integrin-dependent tumor growth and metastasis in NSCLC, as ADAM15 is the only member of the metalloproteinase family located on the cell membrane that contains an RGD fragment in the disintegrin domain. Being expressed by NSCLC cells, the αvβ3 integrin also interacts with the RGD motif of ADAM15, thereby promoting cell proliferation through the FAK signaling pathway [46].

Hynes R.O., in his fundamental article [47], has noted that the bidirectional signaling of integrins contributes to the association between extracellular and intracellular matrix components. Intracellular signal transmission mechanisms may depend on the type of heterodimeric complex, the type of cell, the type of activating factor, and the components of the extracellular matrix [9]. Integrins behave as allosteric bidirectional signaling molecules because intracellular signaling pathways have an impact on specific inner proteins to promote talin binding to the cytoplasmic domain of the integrin β subunit and thus contribute to the integrin’s conformational changes and extrinsic ligand binding, which, in turn, triggers the integrin association with the actin cytoskeleton via talin [48]. Ligand-bound integrins via talin, kindlin, and, probably, other cytoskeletal proteins cooperate with the actin system, leading to integrin clustering and the following activation of FAK and SRC family kinases (SFKs). This enhances significant pro-mitogenic and pro-survival signaling pathways, as well as their transcriptional outputs. These include the Ras-ERK, phosphatidylinositol 3-kinase (PI3K)/AKT, and Yes-associated protein (YAP)/transcriptional coactivator with PDZ-binding motif (TAZ) signaling pathways [49].

The components of the extracellular matrix can also mediate changes in integrin-dependent cellular outside–in signaling. Moreover, the nature of the changes in intracellular processes and the involvement of intracellular effector systems may vary depending on the type of cells and their localization and the type of matrix component predominant in the tissue.

Many growth factors interact with the ECM, and the spatial arrangement of integrins and growth factor-binding sites within the ECM allows for the simultaneous engagement of their corresponding receptors on the plasma membrane. In addition, galectins and specialized plasma membrane domains such as caveolin microdomains regulate the formation of integrin and receptor tyrosine kinase (RTK) signaling complexes and the activation of such signaling proteins as Fyn and Yes through the activation of Shc, a signaling adaptor protein [50].

Integrins play a vital role in providing control over the activity of cytokines and growth factor receptors, thereby coordinating various proliferative, regenerative, and repair processes. To achieve this, integrins and RTKs must be activated in a coordinated manner to ensure optimal signaling through the Ras-ERK and PI3K-AKT pathways, which promote cell survival and proliferation and, under adverse conditions, are able to maintain oncogenic activity. It was demonstrated that integrins participate in the activation of SFKs, which, in turn, induces the phosphorylation of the P-loop of RTKs and its activation [51]. Additional mechanisms contributing to the integration of integrin and RTK signaling include the activity of the inositol phosphatase SHIP2, which acts as a scaffolding protein to enable the recruitment of fibroblast growth factor receptors (FGFRs) to focal adhesions. This promotes the activation of SFKs and the hyperphosphorylation of FAK2, which, in turn, contribute to the prolonged activation of ERK in response to fibroblast growth factors (FGFs). Moreover, integrins and growth factor receptors contribute to activate key signaling molecules, such as Shc, PI3K, Rac, and MEK. This cooperation leads to the optimal activation of various targets downstream, including AP-1 (c-Jun/c-fos), the mammalian target of rapamycin (mTOR) [52].

mTOR contributes to cell survival and cell proliferation, while AP-1 (c-Jun/c-fos), in turn, maintains cell migration, invasion, and proliferation. In normal cells, these major signaling pathways are limited by strong negative-feedback mechanisms, which act as a barrier to tumor development. However, during tumor transformation and progression, the hyperactivation of these potential oncogenic pathways occurs, leading to a disruption of the inhibitory effects that ordinarily control their overactivation (Figure 2) [53].

Numerous intracellular systems can be involved in the process of integrin-mediated intracellular signaling, including FAK, Ras, RhoGTFases, ERK (MAPK), and PI3K/AKT/mTOR [9]. For integrin avβ8, particularly, McCatry et al. described three main intracellular signaling pathways: protein tyrosine phosphatase PTP-PEST, involved in focal adhesions; interaction with RhoGDI1, involved in the regulation of Rho GTPase signaling; the β8 integrin cytoplasmic domain, which can link to the actin cytoskeleton through interactions with Band 4.1 proteins [54].

It has been hypothesized that certain integrins may contribute to tumorigenesis, whereas others may not have such an effect, or may even suppress it [49]. This function is likely due to the ability of integrins to interact with oncogenic RTKs.

The ability of the avβ3 integrin to interact with the platelet-derived growth factor β (PDGF-β) receptor and subsequently enhance its signaling capacity in PDGF-overproducing glioblastomas allows for the consideration of avβ3 as a positive regulator of oncogenesis [55]. This mechanism of action is likely due to the integrin’s ability to induce the phosphorylation of the P-loop of RTKs, thereby supporting their activation. Conversely, activated RTKs may interact with the intracellular domain of the β4 subunit or signaling adaptors associated with avβ3, thereby sustaining the amplification of their signal output. During genetic research, it has been discovered that ErbB2, an RTK protein abnormally increased in HER2-positive breast cancer, interacts with integrin β4. This interaction leads to the impaired adhesion and polarization of the epithelial cells, as well as the promotion of invasion and excessive cell proliferation during breast tumor development [49].

Other integrins may have both supportive and suppressive effects on tumorigenesis, independently of RTK signaling. For instance, avβ5, avβ6, and avβ8 integrins, which are involved in the activation of TGF-β, can inhibit cell proliferation in tumor cells that retain an intact cytostatic response to TGF-β. However, in other contexts, these integrins can induce invasion and metastasis. It has been established that mutations in the TGF-β target genes *CDKN2B* and *CDKN1A* impair TGF-β’s cytostatic function and enhance TGF-β signaling, leading to the implementation of its pro-invasive mechanisms [56].

In general, it should be considered that the direction of changes in the pro- and anti-oncogenic effects of integrins can depend on a wide range of factors, primarily the type of cell they are located in, the concentration of endogenous ligands, and the regulation of cellular responses, which can vary depending on an individual’s immune system status and overall comorbid conditions (such as alcohol abuse, smoking, or living or working in polluted environments), which create additional risks. Integrins represent just one component in the multifaceted carcinogenic process and the equally intricate fibrotic process.

## 5. Drug Strategies for Inhibiting αv Integrins

**Figure 3:**
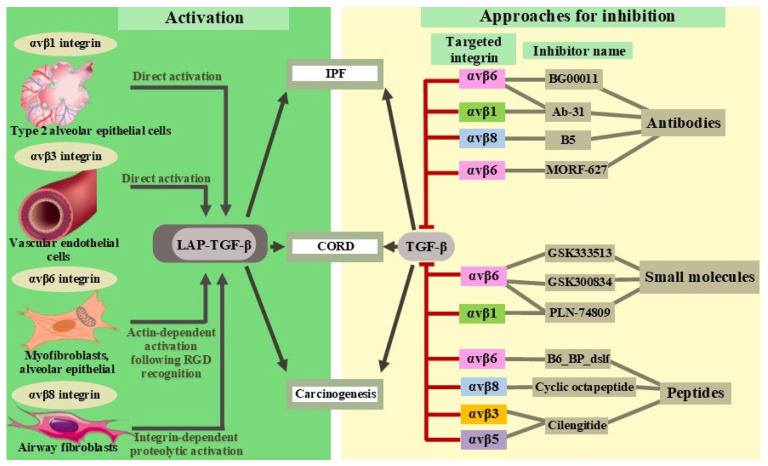
An overview of the various activities of integrins and their potential inhibitors. TGF-β—transforming growth factor-β; LAP-TGF-β—latency-associated peptides in complex with TGF-β; IPF—idiopathic pulmonary fibrosis; CORD—chronic obstructive pulmonary disease; BG00011, Ab-31, B5, MORF-627, B6_Bp_dslf, GSK3335103, GSK3008348, PLN-74809, Cyclic octapeptide, Cilengitide—potential inhibitors of integrins, which are in different stages of clinical trials.

The development of drugs based on integrin inhibitors started in the 1990s, leading to the emergence of a new class of antiaggregants, inhibitors of integrin αIIbβ3 (GPIIb/IIIa). According to Pang et al., more than 100 molecules targeting members of the integrin family are currently being developed [9]. This includes information about the development of inhibitors for the αv integrin with antifibrotic effects (Figure 3).

Currently, there are 561 active clinical trials (according to the ClinicalTrials.gov database) that are relevant to idiopathic pulmonary fibrosis (IPF). An analysis of the data presented on drug and non-drug therapy for IPF has shown that most drugs whose actions target αv integrins have not yet progressed to phase III. Consequently, it is premature to reach a final conclusion regarding their safety and efficacy.

Antibody-based therapies represent a promising treatment strategy for various pathologies, although attempts to use antibodies against αv integrins have failed in certain cases. In a phase IIb, randomized, double-blind, placebo-controlled trial (NCT03573505), BG00011, a monoclonal IgG1 antibody targeting the αvβ6 integrin, was administered subcutaneously to IPF patients. The study was terminated due to safety concerns. The progression of fibrosis as assessed by CT scan was observed in patients receiving BG00011 and in the placebo group. The improvements in the respiratory function measures were not observed in patients receiving BG00011 compared to those receiving the placebo, and patients treated with BG00011 generally had a worse outcome after 26 weeks of treatment. The median duration of BG00011 therapy was 17 weeks, and Raghu et al. state that four deaths were related to respiratory abnormalities, including pneumonia, bronchitis, and acute IPF exacerbation [57].

The Ab-31 antibody effectively blocked the binding of αv integrins to a wide range of ligands containing the arginyl–glycyl–aspartic acid (RGD) sequence and inhibited cell adhesion by suppressing both latent transforming growth factor-β (TGF-β) activation and smooth-muscle α-actin expression induced by activated TGF-β in lung fibroblasts in IPF patients. Moreover, Ab-31 bound firmly to both human αvβ1 and αvβ6 integrins and the corresponding integrins in mice [58].

Airway remodeling in chronic obstructive pulmonary disease (COPD) caused by inflammation and fibrosis depends on not only the αvβ6 integrin but also the αvβ8 integrin. The increased expression of this integrin in airway fibroblasts in patients with COPD makes it an attractive therapeutic target [53]. An optimized antibody to human αvβ8, named B5, inhibited TGF-β activation in transgenic mice expressing only the human and not the murine *ITGB8* gene, which encoded β8. B5 blocked the fibrotic inflammatory response induced by tobacco smoke, cytokines, and allergens by inhibiting TGF-β activation. It has also been demonstrated that the mechanism of action of B5 is related to blocking the low-affinity bent–closed conformation of αvβ8 [59].

Experiments with oral integrin inhibitors have not always been successful at reaching the clinical trial stage. The oral selective inhibitor of the αvβ6 integrin MORF-627, which stabilizes the bent–closed conformation of the αvβ6 integrin, has been shown to prevent changes in the cell structure with delayed fibrosis development. However, in a 28-day safety study on a group of Javan macaques, the administration of MORF-627 resulted in the induction of epithelial proliferative changes in the bladder in two of six animals. Meanwhile, MORF-627 was found to be nongenotoxic in in vitro experiments. Bladder tumors were highly unusual, considering the lack of genotoxicity of the compound, the short latency period, and the absence of concurrent chronic damage to the bladder mucosa. One potential explanation for this phenomenon of the inhibition of the αvβ6 integrin is attributed to the different signaling role of TGF-β in cell cycle regulation and carcinogenesis. The dysregulation of the TGF-β signal can play a significant role in carcinogenesis, acting as either a suppressor or stimulator of tumors [60,61]. According to the study by Wendt et al., “this ‘TGF-β paradox is not always explicable” [61,62]. The development of the drug has been suspended, but MORF-627 may serve as a tool for further studies of αvβ6 integrin biology [63,64].

The de novo-developed compound B6_BP_dslf is a small (<75 amino acids) protein with high selectivity for the αvβ6 integrin. The action of the compound is comparable to the BG00011 antibody, which was excluded from further development due to its potential to exacerbate disease and cause mortality. B6_BP_dslf binds to the canonical ligand-binding site in the α/β subunit cleft, stabilizing the open conformation of αvβ6. The therapeutic efficacy of this miniprotein was evaluated in a bleomycin-induced lung fibrosis model in mice. B6_BP_dslf stopped the progression of fibrosis, as measured by microcomputed tomography and a visual assessment of stained lung slices (Ashcroft scale). The properties of B6_BP_dslf remain unchanged after aerosol administration, making it suitable for inhalation delivery and limiting the systemic effects [65].

GSK3335103, an oral αvβ6 integrin inhibitor, suppresses TGF-β activation in human IPF lung tissues and mouse models of bleomycin-induced pulmonary fibrosis. The inhibition of TGF-β activation resulted in decreased collagen deposition in mice with pulmonary fibrosis [66]. Hryczanek H.F. et al. described the simplified chemical synthesis of two potent αvβ integrin inhibitors by modifying the GSK3335103 molecule. Both compounds have good bioavailability when administered orally [67].

Compound GSK3008348, an inhaled αvβ6 integrin inhibitor, is the first inhibitor in its class that has reached clinical development. GSK3008348 binds with high affinity to αvβ6 integrins in human lungs with IPF and reduces TGF-β signaling [68]. The safety and good tolerability of GSK3008348 has been demonstrated in phase I trials in healthy volunteers [69]. A phase Ib trial has also been completed, proving the targeted interaction of the compound with αvβ6 integrins in IPF patients. GSK3008348 was well tolerated, and no serious adverse events or clinically significant abnormalities were observed. In a study conducted by Maden et al. [69], GSK3008348, developed as an aqueous solution, was administered using a nebulizer for 10 min. The direct effect of the aerosol on the lungs in combination with resorptive effects increases the potential of the drug to reach all areas of the lungs over a long period at lower doses than with other methods of administration. At the same time, the use of GSK3008348 in doses up to 3000 micrograms did not lead to the development of local and systemic side effects. In the same study, some pharmacokinetic parameters of the compound were determined, in particular, the optimal dosage regimen of two times a day [70]. However, it may also present challenges for using a nebulizer, such as the conditional unpredictability of the dosage, as some of the medication may settle on the plastic components of the device. Additionally, breathing for 10 min may be physically difficult for patients with IPF. The effect of integrin inhibitors may manifest locally through poorly predictable side effects on the mucous membranes and microflora, whose impacts have not been fully studied. The probability of the occurance of such adverse effects can be assessed by conducting at least some of the toxicological studies in primates.

PLN-74809, a dual inhibitor of αvβ6 and αvβ1 integrins, demonstrated high efficacy on human lung precision sections and in a mouse model of bleomycin-induced pulmonary fibrosis. The dual inhibition of αvβ6/αvβ1 with PLN-74809 reduced the type I collagen gene expression by approximately 50% at a concentration of 2 nM in human and mouse lung fibrotic sections. The antifibrotic activity of PLN-74809 has also been confirmed in vivo. The dose-dependent inhibition of pulmonary Smad3 phosphorylation and collagen deposition has been observed in lung tissue and bronchioalveolar lavage cells in mice. The Smad3 protein functions in the TGF-β signaling pathway and transmits signals from the cell surface to the nucleus, regulating cell proliferation [44,71]. In the INTEGRIS-IPF (phase IIa) trial, the dual inhibitor PLN-74809, named bexotegrast, demonstrated a positive safety and tolerability profile when administered once a day at doses of 40 mg, 80 mg, 160 mg, or 320 mg. Compared to the placebo, bexotegrast was associated with a reduction in fibrosis-related biomarkers [72]. In the phase II NCT04072315 trial, bexotegrast demonstrated a dose-dependent increase in the percentage of αvβ6 receptor occupancy in patients with IPF. However, there was a lack of data regarding the side effects associated with treatment [73]. A phase II, randomized, double-blind, placebo-controlled, dose-varying trial to evaluate the efficacy and safety of bexotegrast (PLN-74809) in the treatment of IPF was launched in 2023 and will end in September 2025. Bexotegrast has also been shown to be effective in renal fibrosis. Administration of the drug to mice with a model of renal fibrosis induced by an adenine diet and to mice with unilateral ureteral obstruction dose-dependently delayed the progression of fibrotic transformation. In renal fibrosis models, bexotegrast, via the dual inhibition of αvβ6/αvβ1, disrupts the FAK/Src/AKT/β-catenin pathway function [74].

The development and potential medical application of a cyclic octapeptide as a highly selective low-molecular-weight ligand for αvβ8 has also been reported. At low affinity, the compound has demonstrated the marked inhibition of the αvβ8 integrin. The use of the peptide with fluorescent and radioactive tags allows for the visualization of αvβ8-positive cells and tissues [75].

Cilengitide is a cyclic pentapeptide that mimics the binding motifs of some ECM proteins, such as fibronectin and vitronectin. The agent inhibits ligand binding to αvβ3, αvβ5, and α5β1 integrins. In mouse models of glioblastoma, the antitumor effect of cilengitide has been demonstrated, as well as its ability to inhibit angiogenesis. In a study by Ritzenthaler J.D. et al., the treatment of cultured fibroblasts with cilengitide resulted in the suppression of fibrotic process development, which was confirmed by histologic analysis (Ashcroft score), the number of cells in bronchoalveolar lavage, and the hydroxyproline content. However, daily injections of cilengitide in mice with bleomycin-induced lung injury did not improve the course of fibrosis (Table 2) [76].

## 6. Conclusions

The inflammatory process can trigger the tumor transformation of cells. Chronic obstructive pulmonary disease (COPD) and lung cancer often occur as comorbid diseases. According to some data, 40–70% of patients simultaneously suffer from COPD and lung cancer [77]. The association of COPD and lung cancer can lead to the unstoppable rapid progression of the latter [78]. Infectious lung diseases can also be combined with lung cancer [79]. Thus, it can be assumed that the phenomenon may be based on a violation of antitumor immunity as a result of infectious or non-infectious inflammation.

The treatment of fibrotic lung diseases, including idiopathic pulmonary fibrosis (IPF) and lung cancer as a complication, remains challenging, as the currently available treatment options are ineffective at halting the progression of the condition. Drug development efforts have focused on attenuating fibrotic responses by phosphodiesterase activity inhibition, affecting the autotaxin-receptor lysophosphatidic acid signaling pathway and blocking the αvβ6 and αvβ1 integrin activity [80]. One of the therapeutic strategies involves targeting the transforming growth factor-β1 (TGF-β1) signaling pathway, which is a key mediator and central profibrotic cytokine in multiple-organ fibrosis. Elevated TGF-β1 levels have been observed in patients with IPF, and its expression is concentrated in fibroblastic foci and associated with sites of active fibrosis and collagen biosynthesis [81]. TGF-β has been proven to regulate the epithelial–mesenchymal transition in lung cancer [3]. Integrins play a crucial role in TGF-β1 activation in pulmonary fibrosis. Recent studies have demonstrated that the knockout of the αv subunit gene in myofibroblasts effectively reduces TGF-β1 activation and prevents fibrosis in the liver, kidney, and heart, as well as bleomycin-induced lung fibrosis in mouse models [82]. Therefore, integrins have become more prominent as potential therapeutic targets due to their transmembrane location and high availability for specific inhibitors.

Based on the structural features of integrins, it might be suggested that the potential drug targets should include the region between the I and hybrid domains near the α7-helix in the β subunit, the invariant isoleucine residue in the I domain of the α subunit, the cytoplasmic Glu-Phe-Phe-Lys-Arg (GFFKR) sequence of the α subunit or, in general, the salt bridge it forms with the cytoplasmic part of β subunits, and the Ca^2+^ ion in the adjacent sites to metal ion-dependent adhesion site (ADMIDAS) region of the β subunit (Figure 1). According to some investigations, it is essential to consider that the potential inhibitor should be the closure-stabilizer of the integrin molecule, as fixing the open conformation leads to failures in the clinical research stage [83].

The research of Lin et al. [83] analyzed a wide range of existing inhibitors that have progressed to clinical trials, specifically integrin αIIbβ3. The patterns obtained from this analysis were then applied to other integrins, particularly to α4β1. The study focused on identifying the reasons why these inhibitors of αIIbβ3 have failed in stage III of five clinical studies in the field. After receiving negative results from these studies, concerns regarding the feasibility of developing integrin inhibitors have been raised. To some extent, the negative outcomes of the clinical trials can be explained by the fixation of integrin αIIbβ3 in an extended–open conformation. The cause may be the development of antibodies to “pre-existed or ligand-induced binding sites (LIBS)” [84]. Furthermore, the development of αIIbβ3 antagonists was stopped prior to the binding of their first structures to αIIbβ3, which were all with LIBS inducers. The subsequently developed LIBS-inducing integrin αvβ3 inhibitor also failed in clinical trials in cancer, and it has been suggested that it is agonistic [85].

Lin et al. [83] propose that the fixation of an integrin in a bent–closed conformation may not induce the production of antibodies, as the binding sites of the integrin molecules, which could potentially provoke antibody formation, may not be exposed. Moreover, Lin et al. [83] have analyzed the role of magnesium and manganese ions, as well as the presence of water molecules. They have described the possible chemical structure of a small integrin inhibitor molecule and its interaction with key amino acids within the integrin molecule from the perspective of molecular dynamics. Researchers have demonstrated that compounds with the property of integrin inhibition can fix integrins in a bent–closed conformation when they possess nitrogen-containing and carboxylic acid groups in their structures. Thus, the commercially successful implementation into clinical practice of inhibitors of integrin αIIbβ3, such as tirofiban and eptifibatide, stabilize the bent–closed state by mimicking the effect of Arg in the arginyl–glycyl–aspartic acid (RGD) fragment, forming hydrogen bonds with the residue of Asp-224 in the α-chain. At the same time, a carboxyl group must be presented in the molecule of a potential inhibitor in order to simulate the binding of Asp, which is part of the RGD sequence that coordinates the metal ion-dependent adhesion site (MIDAS) metal ion in the β-chain. Additionally, for the conversion of an open structure to a bent–closed structure, it is essential that there is a bridge between the sidechain of the MIDAS residue Ser-123 and a MIDAS metal ion [79]. Therefore, a potential inhibitor can be recognized similar to an endogenous ligand that contains an RGD fragment. However, in forming the extended–open and bent–closed conformations of other heterodimeric complexes, the same Asp and Ser residues may be involved, but they tend to be located in different positions due to differences in the subunit structure (Table 1).

In this regard, it is hypothesized that the fixation of integrins in a bent–closed conformation is facilitated by the binding of low-molecular-weight inhibitors to three specific points, as shown in Figure 1. This hypothesis is currently a working assumption and requires further experimental validation.

Discussing the transfer of basic knowledge into clinical practice, it can be stated that the most significant issue tends to be the development of medicinal agents aimed at integrins for the treatment of fibrotic and tumorous diseases. In this instance, it would be overly optimistic to discuss the possibility of using integrin inhibitors as monotherapy for ILF, COPD, and, especially, tumor diseases. Rather, it is more likely that, in the management of tumor diseases and their combination with fibrotic tissue lesions, integrin inhibitors may be employed as part of a complex therapeutic regimen to enhance the efficacy of other drugs. However, it is worth noting that some substances that have progressed to the clinical trial phase may not meet the desired outcomes due to unforeseen, unacceptable toxicity. Nevertheless, these substances may still be utilized in the future as high-affinity components in the development of diagnostic tools (histological investigations). In the case of clinical trial failure due to insufficient efficacy despite well tolerability, compounds targeting integrins may be used as a means of delivering drugs to tumor cells in the form of conjugated complexes. Similar compounds could also be used to develop methods for imaging integrins in tumor tissues in vivo (CT, PET). According to Pang et al., there were four ongoing clinical trials observed for imaging agents, targeting αv integrins, at the beginning of 2023 [9].

It is clear that the issue of creating medicinal products that specifically target integrins on strictly defined cell types, such as fibroblasts, requires further research. It is essential to selectively suppress αv-dependent signaling in fibroblasts while maintaining its function in other tissues. This requires a careful study of the pharmacodynamics and pharmacokinetics of targeted drugs to ensure that they do not block the function of αv in all tissues. When developing drugs, it may be necessary to target the active substance to assure its binding to the membrane of fibroblasts and not to other cell types. The current clinical trial drugs do not specifically target fibroblasts for binding. This challenge also requires the focused efforts of researchers developing new low-molecular-weight inhibitors. When translating preclinical research, it is essential to take into consideration potential species variations in the localization, expression, and affinity of integrins within human and animal tissues, as well as any alterations in the presence of abnormal forms of integrins on various tumor cell lines. Another significant issue that requires attention is the possibility of developing specific drug forms or delivery systems for these compounds to target specific organs in order to reduce their overall toxic effects on the human body. Thus, a special inhaled form of the compound GSK3008348 was developed, and indeed, no local or systemic toxic effects were observed during its use [70]. Hypothetically, the development of liposomal drug forms targeting other fibroblast membrane receptors may be beneficial for delivering inhibitors to fibrotic or tumor sites. Additionally, one possible targeted method of drug delivery to tumor cells might be considered the development of drug forms containing liposomal nanoparticles [86]. A similar strategy is being applied in the development of liposomal forms of drugs with antifibrotic activity, such as nintedanib [87,88]. However, this approach is currently in the experimental stage and has not yet been developed for integrin inhibitors. This information is crucial for the investigation of both the antifibrotic and antitumor properties of small-molecule inhibitors.

Due to integrins’ mechanism of action, the same molecules may be involved in different pathological processes. Specifically, αv integrins have a role in the development of pulmonary fibrosis associated with COPD and IPF, while the excessive activation of TGF-β by integrins initiates tumor cell transformation. Thus, it might be assumed that inhibiting overexpressed αv integrin variants may contribute not only to the regression of pulmonary fibrosis but also simultaneously to a reduced risk of lung cancer.

## Figures and Tables

**Table 1:** Structural differences among β subunits. MIDAS—metal ion-dependent adhesion site; ADMIDAS—adjacent to the MIDAS; SyMBS—synergistic metal ion-binding site; NPxY motif—Asn-Pro-x-Tyr. The data were obtained from the open-access resource the Research Collaboratory for Structural Bioinformatics Protein Data Bank (RCSB PDB). The information on the β4 and β5 ectodomains was not presented in the RCSB PDB.

Subunit	Structural Features	Localization
β1	Mg^2+^ of MIDAS is associated with Ser132, Ser134, and Glu229.	Widely spread
β2	Ca^2+^ of MIDAS is associated with Ser116, Asp119, Asp120, and Glu325.	Leucocytes
β3	Ca^2+^ of MIDAS is associated with Ser123, Asp126, Asp127, and Met335.	Platelets and macrophages
β4	The heaviest subunit, as its cytoplasmic domain consists of approximately 1000 amino acid residues.	Epithelial cells
β5	Not found	Brain and blood cells
β6	Mg^2+^ of MIDAS is associated with Asp140, Ser142, Glu240, and Asp271.	Epithelial cells
β7	Two NPxY motifs in cytoplasmic part for binding with talin; Mg^2+^ in MIDAS is associated with Ser160; two Ca^2+^ in ADMIDAS and SyMBS associated with Pro257, Asp197, Asp255 and Glu372, Asp166, and Ser162.	Immune cells (NK-cells, B-plasma cells, eosinophils, lymphocytes)
β8	Does not interact with talin; does not have ADMIDAS with Ca^2+^; Mg^2+^ of MIDAS is associated with Ser116, Ser114, Asp219, and Glu212; Ca^2+^ of SyMBS is associated with Asp151, Asn207, Asp209, Pro211, and Glu212.	Genitourinary system (kidneys, placenta, uterus, ovaries)

**Table 2:** Summary of potential inhibitors targeted to αv integrins.

Integrin Inhibitor	Drug Type	Targeted Integrin	Study Status	Reason for Failure
BG00011	Antibody	αvβ6	IIb (terminated)	Four deaths related to respiratory complications
Ab-31	Antibody	αvβ1, αvβ6	Preclinical stage	
B5	Antibody	αvβ8	Preclinical stage	
MORF-627	Small molecule	αvβ6	Preclinical stage	Bladder epithelium hyperplasia
B6_BP_dslf	Peptide	αvβ6		
GSK3335103	Small molecule	αvβ6	Preclinical stage	
GSK3008348	Small molecule	αvβ6	I (terminated)	The reasons are not clear
PLN-74809	Small molecule	αvβ6, αvβ1	II (ongoing)	Will end in September 2025
Cyclic octapeptide	Peptide	αvβ8		
Cilengitide	Peptide	αvβ3, αvβ5, α5β1	II (terminated)	Did not improve the course of fibrosis in mice with bleomycin-induced lung injury

## Data Availability

No new data were created or analyzed in this study. Data sharing is not applicable to this article.

## Abbreviations

The following abbreviations are used in this manuscript:

IPF	Idiopathic lung fibrosis
COPD	Chronic obstructive pulmonary disease
TGF-β	Transforming growth factor-β
ECM	Extracellular matrix
WHO	World Health Organization
RGD	Arginyl–glycyl–aspartic acid
GFFKR	Glu-Phe-Phe-Lys-Arg
MIDAS	Metal ion-dependent adhesion site
Hyb	Hybrid domain of β subunit
PSI	Plexin–semaphorin–integrin
EGF	Epidermal growth factor
ADMIDAS	Adjacent sites to metal ion-dependent adhesion site
SyMBS	Synergistic metal ion-binding site
NPxY	Asn-Pro-x-Tyr
NSCLC	Non-small-cell lung cancer
LAP	Latency-associated peptide
MMP2	Matrix metalloproteinase 2
MMP9	Matrix metalloproteinases 9
ALK 5	Activin-like kinase 5
CCL2	C-C motif chemokine ligand 2
NF-kB	Nuclear factor kappa-light-chain-enhancer of activated B cells
FAK	Focal adhesion kinase
AKT	Protein kinase B
MMP12	Matrix metalloproteinase 12
PYK2	Proline-rich tyrosine kinase 2
STAT3	Activator of transcription 3
LARP6	La-related protein 6
ADAM15	Metalloproteinase 15
SFKs	SRC family kinases
ERK	Extracellular signal-regulated kinase
PI3K	Phosphatidylinositol 3-kinase
YAS	Yes-associated protein
TAZ	Transcriptional coactivator with PDZ-binding motif
RTK	Receptor tyrosine kinase
FGFR	Fibroblast growth factor receptor
FGFs	Fibroblast growth factors
mTOR	Mammalian target of rapamycin
PDGF β	Platelet-derived growth factor β
LIBS	Ligand-induced binding site
